# Physicians’ attitudes toward medical and ethical challenges for patients in the vegetative state: comparing Canadian and German perspectives in a vignette survey

**DOI:** 10.1186/1471-2377-14-119

**Published:** 2014-06-05

**Authors:** Katja Kuehlmeyer, Nicole Palmour, Richard J Riopelle, James L Bernat, Ralf J Jox, Eric Racine

**Affiliations:** 1 Institute of Ethics, History and Theory of Medicine, University of Munich, Munich, Germany; 2 Neuroethics Research Unit, Institut de recherches cliniques de Montréal, Montréal, Canada; 3 Department of Neurology and Neurosurgery, McGill University, Montréal, Canada; 4 Neurology Department, Dartmouth-Hitchcock Medical Center, Lebanon, USA; 5 Department of Medicine and Department of Social and Preventive Medicine, Université de Montréal, Montréal, Canada

**Keywords:** Vegetative state, End-of-life, Prognosis, Ethics

## Abstract

**Background:**

Physicians treating patients in the vegetative state (VS) must deal with uncertainty in diagnosis and prognosis, as well as ethical issues. We examined whether physicians’ attitudes toward medical and ethical challenges vary across two national medical practice settings.

**Methods:**

A comparative survey was conducted among German and Canadian specialty physicians, based on a case vignette about the VS. Similarities and differences of participants’ attitudes toward medical and ethical challenges between the two samples were analyzed with non-parametric tests (Mann-Whitney-*U*-Test).

**Results:**

The overall response rate was 13.4%. Eighty percent of all participants correctly applied the diagnostic category of VS with no significant differences between countries. Many of the participants who chose the correct diagnosis of VS attributed capabilities to the patient, particularly the ability to feel pain (70%), touch (51%) and to experience hunger and thirst (35%). A large majority of participants (94%) considered the limitation of life-sustaining treatment (LST) under certain circumstances, but more Canadian participants were in favor of always limiting LST (32% vs. 12%; Chi-square: p < 0.001). Finding long-term care placement was considered more challenging by Canadian participants whereas discontinuing LST was much more challenging for German participants.

**Conclusions:**

Differences were found between two national medical practice settings with respect to physicians’ experiences and attitudes about treatment limitation about VS in spite of comparable diagnostic knowledge.

## Background

In the last decade the development of diagnostic criteria for disorders of consciousness, particularly the vegetative state (VS, or UWS: unresponsive wakefulness syndrome [1]) and the minimally conscious state (MCS) [2], has reflected a more advanced understanding of impaired responsiveness and awareness following brain injury. Diagnostic guidelines remain largely based on the observation of the patient’s behavior: a patient in a VS shows wakefulness without behavioral signs of awareness, such as meaningful reaction to stimuli, whereas a patient in a MCS shows intermittent behavioral signs for awareness, but is incapable of reliable communication [3,4].

Disorders of consciousness are often misdiagnosed [5-8] and cases of “miraculous” late recovery have questioned timelines for determining the irreversibility of the VS [9-11]. Surveys of physicians’ attitudes toward end-of-life decisions and publically discussed legal cases of decisions about withdrawal of artificial nutrition and hydration have shown considerable diversity in attitudes among different healthcare professions and countries [12-17]. To further our understanding of the nature and causes of this diversity with respect to attitudes towards VS and decisions about the limitation of life-sustaining treatment (LST), we undertook a bilateral survey among physicians in Canada and Germany. The survey assessed physicians’ understanding of the VS and elicited their attitudes towards end-of-life issues in two different national medical practice settings. Parts of the survey completed by German neurologists have been previously reported [18]. In this paper, we focus on the comparison between the attitudes of German and Canadian physicians.

## Methods

### Study design

A self-administered questionnaire survey enabled us to target large groups of physicians. In Germany the questionnaire was distributed by e-mail. In Canada, it was converted into a paper-based questionnaire because the emailed survey did not generate sufficient participation (n = 27).

The data were gathered anonymously and participants gave their informed consent. To encourage participation the participants of the German survey were offered an opportunity to take part in a lottery, consisting of six prizes with a total value of € 1500. After three weeks, we sent a reminder and prolonged the participation eligibility period for an additional week. To encourage participation in the Canadian survey, two postcard reminders were sent following the initial invitation.

The study was approved in Canada by the research ethics board of the Institut de recherches cliniques de Montréal, and in Germany by the research ethics committee of the medical faculty at the University of Munich.

### Participants

To include a representative cohort of German neurologists, we contacted the German Society for Neurology, which facilitated the distribution of the survey link. Out of 6673 members, we contacted all 3073 members for whom the society had valid e-mail addresses and invited only physicians to participate.

In the Canadian survey, questionnaires were mailed to participants in their preferred language of correspondence (French or English) as listed in Scott’s Medical Directory. Neurologists, neurosurgeons, emergency medicine and critical care physicians, and physical medicine and rehabilitation specialists registered with the Royal College of Physicians and Surgeons of Canada were included in the cohort. We mailed surveys to 2085 participants.

### Questionnaire

We developed a 37-item questionnaire that was informed by the literature and a previous qualitative study and our study goals [19]. The first part was a case vignette about a patient in the VS (1).

A 33-year-old man had a cardiac arrest with delayed resuscitation 4 months ago. Currently, he shows brainstem and spinal reflex movements, but no sign of purposeful movement. His eyes are open for several hours a day, but do not fixate objects or follow them when they move. He does not react consistently* to verbal commands or questions. Sometimes a delayed stiffening of the legs and grimacing can be observed in reaction to sounds. He can breathe on his own.

In response to the results and comments from the German online survey, the wording of the case was slightly modified in the Canadian print survey. To reduce ambiguity, the sentence “He does not react *consistently* to verbal commands or questions” was changed to “He does not react to verbal commands or questions”. For detailed information on the questionnaire development and content see our previous publication [7]. The questionnaire was translated from English into German and French using backward-forward-translation by native speakers. The German data were gathered within a four-week period from July to August 2011, the Canadian data were gathered within a four-month period from January to May 2012 for the postal survey.

### Statistical analysis

Data from the paper version of the survey were imported into IBM SPSS 19 statistics software. Pearson’s chi-square test was performed to assess differences between categories. For numerical or ordinal data the Mann-Whitney-*U*-Test was applied to compare two groups. Results were considered significant if p < 0.05. P-values are descriptive and were not adjusted for multiple comparisons. The study was powered to detect a 15% difference in the attitudes towards limiting LST between the two countries with a probability of 80%. Participants who failed to correctly diagnose the patient in the case vignette were excluded from further analysis of their attitudes, as it was unclear whether they answered the remaining questions according to the vignette description or according to their inaccurate diagnosis.

## Results

### Samples

The demographic and professional characteristics of the participants in both samples are presented in Table 1. Of the 3073 professionals who were contacted in the German survey, one third (n = 1024) was randomly assigned to receive the VS case. Out of these, 168 participants (16.4%) completed a questionnaire according to the VS case. The cohort of 3073 members of the German Society for Neurology was representative for all members (N = 6673) according to age (members: mean 44, standard deviation (SD) 10, range 25–94, and cohort: mean 45, SD 9, range 25–87) and region of practice, but not gender. A lower percentage of women was invited to participate (28%) than the actual percentage of women in the Society (38%). The sample was representative for the Society according to age (mean 43, SD 9, range 27–80).

**Table 1:** Demographic and professional characteristics of participants (n = 417)

**Variable**	**Canadian sample (n = 249)**		**German sample (n = 168)**	
**No. (%)**				
Gender (n = 395)				
Female	63	(27)	47	(30)
Male	175	(74)	110	(70)
Primary discipline*				
Neurology	114	(46)	161	(96)
Neurosurgery	41	(17)	1	(1)
Rehabilitation medicine	42	(17)	20	(12)
Emergency medicine	35	(14)	-	-
Others (e.g., anesthesiology)	10	(4)	20	(12)
Health care setting*				
In-patient care	185	(74)	115	(69)
Out-patient care	197	(79)	62	(37)
Type of care*				
Acute care	159	(64)	71	(42)
Rehabilitation care	47	(19)	36	(21)
Long-term care	26	(10)	17	(10)
Professional experience with patients in the VS (n = 383)				
0 cases	28	(13)	2	(1)
1-10 cases	123	(55)	94	(60)
> 20 cases	73	(33)	63	(40)
Religious practice (n = 398)				
Practicing religion	99	(41)	92	(58)
Not practicing religion	141	(59)	66	(42)
Spiritual beliefs (n = 392)				
Spiritual beliefs	132	(56)	100	(64)
No spiritual beliefs	104	(44)	56	(36)
**Median; 1**^**st**^**, 3**^**rd **^**quartile (range)**				
Age (years)	53; 41,59	(30 – 81)	43; 37,48	(27 – 80)
Experience (years)	21; 9,30	(<1 – 56)	15; 10;22	(2 – 51)

*Multiple answers permitted.

Of the 2085 physicians contacted in the Canadian survey, 249 physicians (11.9%) completed and returned the questionnaire. Twelve declined to participate, 10 of them indicating that they were not working with patients in the VS. The sample was representative for the cohort according to gender and language. Almost the same percentage of women contacted (n = 564, 27%) responded to the survey (n = 63, 25%). The majority of participants filled out the English version of the questionnaire (n = 192, 77%), while one fifth returned the French version (n = 57, 23%), which matched the distribution of English and French questionnaires (English: n = 1659, 80%; French: n = 426, 20%). Among neurologists, the response rate was slightly higher than in the cohort (46% compared to 39%). A comparable percentage of physical medicine and rehabilitation specialists and neurosurgeons responded (specific response rate 17% compared to 18% in the cohort; and 16% compared to 13% in the cohort respectively). A lower percentage of critical care and emergency medicine specialists (specific response rate 14% compared to 30% in the cohort) returned the survey.

### Application of diagnostic knowledge

Overall 80% (n = 332) of all participants (n = 417) correctly chose the diagnostic category of VS for the patient in the case vignette. In both samples a similar proportion chose the correct diagnostic category (80% in Canada, 79% in Germany; p = 0.66). A comparison between the neurologists in both samples showed analogous results: 82% of the Canadian neurologists (n = 115) compared to 79% of the German neurologists (n = 161) who assigned the correct diagnostic category to the case vignette (Chi-square-test: p = 0.56). The Canadian participants who received the original case vignette in the online survey (n = 27) and those who received the slightly edited case vignette in the paper and pencil survey (n = 222) did not differ significantly in their accuracy rate (81% vs. 78%, Chi-square test: p = 0.73). In both countries a comparable proportion suggested an erroneous diagnosis of MCS for the patient (18% in German and 15% in the Canadian sample). Only a few respondents chose the diagnoses of locked-in-syndrome (2% vs. 1% in the Canadian sample), coma (1% vs. 2% in the Canadian sample), brain death (none vs. 1% in the Canadian sample), or others (e.g., 1% suggesting the patient is in different conditions at the same time).

Most participants expressed a high degree of certainty of their diagnosis (median = 8; 1^st^ quartile = 6; 3^rd^ quartile = 9 on a NRS (0-10)), regardless of correct (87%) or incorrect (70%) diagnosis. Canadian and German participants did not differ significantly in their level of certainty (p = 0.71). We continued our data analysis with the participants who accurately applied the diagnostic knowledge to the case (hereafter referred to as “all groups” = all participants who applied the correct diagnostic knowledge; “Canadian group” = all participants in the Canadian sample who applied the correct knowledge; and “German group” = all participants in the German sample who applied the correct diagnostic knowledge).

### Prognosis and quality of life

Most of the participants estimated the prognosis of the patient’s survival under continuous medical support during the next 6 months to be favorable with a majority predicting good or very good chances for survival (See Table 2) but opinions of Canadian and German physicians showed a different distribution. Assessments of quality of life also differed between the two groups of physicians. Canadian physicians formulated bleaker assessments of quality of life than their German counterpart who also more frequently responded not being able to rate quality of life.

**Table 2:** Prognosis of survival and estimation of the patient’s quality of life

	**No. (%)**			
	**All**	**Canadian**	**German**	***p *** **value**
	**Groups (n = 332)**	**group (n = 200)**	**group (n = 132)**	
Prognosis of survival (n = 329)				p = 0.049
None (0%)	1 (0.3)	1 (1)	-	
Minimal (<10%)	7 (2)	5 (3)	2 (2)	
Very small (10-25%)	18 (6)	9 (5)	9 (7)	
Small (26-50%)	42 (13)	24 (12)	18 (14)	
Good (51-75%)	122 (37)	67 (34)	55 (42)	
Very good (76-90%)	95 (29)	57 (29)	38 (29)	
Excellent (>91%)	30 (9)	27 (14)	3 (2)	
Certain (100%)	2 (1)	-	2 (2)	
Not able to rate	12 (4)	8 (4)	4 (3)	
Estimation of the patients quality of life (QoL) (n = 327)				p < 0.001
No QoL	109 (33)	91 (46)	18 (14)	
Extremely low (0)	91 (28)	58 (29)	33 (25)	
1	46 (14)	28 (14)	18 (14)	
2	30 (9)	11 (6)	19 (15)	
3	5 (2)	2 (1)	3 (2)	
4	2 (1)	-	2 (2)	
>4	-	-	-	
Not able to rate	44 (14)	7 (4)	37 (29)	

QoL = Quality of life.

### Assessment of the patient’s capabilities

Although having diagnosed the patient in the case vignette as VS, participants attributed residual capabilities to VS patients such as feeling pain, feeling touch, experiencing hunger/thirst, smelling odors, and tasting flavors of food/drinks (see Table 3). More German than Canadian participants attributed such capabilities to VS patients.

**Table 3:** Attribution of capabilities of the patient in the vegetative state

**Agreement**	**No. (%)**				
	**All groups**	**Canadian**	**German**	D (%)*	***p *** **value**
	**(n = 332)**^**1**^	**group**^**1**^	**group**^**1**^		
		**(n = 200)**	**(n = 132)**		
Feeling pain	232 (70)	130 (65)	102 (77)	(12)	p = 0.017
Feeling touch	168 (51)	79 (40)	89 (67)	(27)	p < 0.001
Experiencing hunger/thirst	117 (35)	57 (29)	60 (46)	(17)	p = 0.002
Smelling odors	94 (28)	48 (24)	46 (35)	(11)	p = 0.032
Tasting flavors of food/drinks	69 (21)	31 (16)	38 (29)	(13)	p = 0.003
Experiencing dreams	70 (21)	23 (12)	47 (36)	(24)	p < 0.001
Having emotions	63 (19)	17 (9)	46 (35)	(26)	p < 0.001
Having thoughts	48 (15)	18 (9)	30 (23)	(14)	p < 0.001
Being aware of themselves	24 (7)	12 (6)	12 (9)	(3)	p = 0.287
Recognizing their name	23 (7)	8 (4)	16 (12)	(8)	p = 0.005
Recognizing people	23 (7)	6 (3)	17 (13)	(10)	p = 0.001
Remembering experiences	22 (7)	5 (3)	17 (13)	(10)	p < 0.001
Being aware of surroundings	20 (6)	12 (6)	8 (6)	(0)	p = 0.982
Having sexual desires	19 (6)	2 (1)	17 (13)	(12)	p < 0.001
Understanding what others say	16 (5)	5 (3)	11 (8)	(5)	p = 0.015
Storing new information	14 (4)	4 (2)	10 (8)	(6)	p = 0.013
Interacting with others	14 (4)	3 (2)	11 (8)	(6)	p = 0.002
Expressing desires	5 (2)	2 (1)	3 (2)	(1)	-

^1^ Those who correctly diagnosed the patient; when expected frequencies in the respective cells were <5 the chi-square-test was not conducted; *D (%) = Difference (% German group –% Canadian group).

### Attitudes towards LST

**Figure 1:**
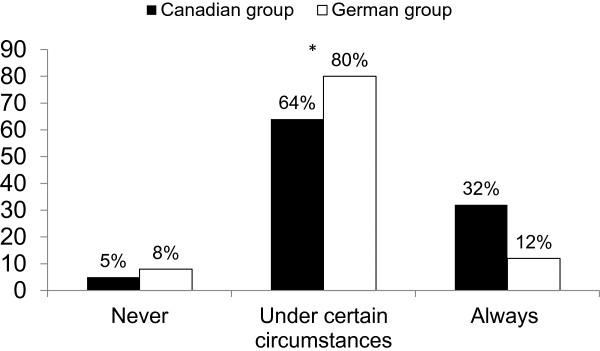
**Attitudes towards the limitation of LST.** Attitudes of participants who assigned the correct diagnosis to the vignette towards the question: “In the prior case life-sustaining treatment should be limited…?” Overall the differences between the two groups were significant (Chi-square test: p < 0.001). N = 332, Canadian group (N = 195), German group (N = 131); Missing data: 6; numbers may not add up to 100 due to rounding.

The Canadian participants were more likely to favor limiting LST than the German participants (see Figure 1). The willingness to limit LST in specific circumstances (e.g., patient’s will is opposed to LST; patient suffers from fatal disease; surrogate refuses consent to LST; no chance of recovery of consciousness; no improvement > 1 year) varied considerably (see Additional file 1: Table S1). However, there was a trend: Canadian participants were more likely than German participants to favor the limitation of LST in almost all circumstances.

### Treatment measures

The willingness to limit specific life-sustaining treatment measures showed a similar pattern in both samples. Overall, 86% would limit cardiopulmonary resuscitation, and the same percentage would do so with regard to intubation and mechanical ventilation. The limitation of hemodialysis and hemofiltration was considered by 80%; the performance of a surgery by 65%. Antibiotic treatment would be limited by 44%, artificial nutrition by 37% and artificial hydration by 30%.

Although there were several differences between the Canadian and the German group in the participants’ attitudes towards treatment limitation, there was only one statistically significant difference in their attitudes towards the limitation of specific treatment measures: 36% of Canadian participants, compared to 23% of German participants (p < 0.05) were willing to limit artificial hydration.

### Appraisal of ethical challenges

The appraisal of previously identified ethical challenges is displayed in Table 4 (e.g., determining patient’s wishes; accompanying family members in decisions; finding long-term care; making prognosis and predicting recovery). All such challenges were ranked differently in the two samples.

**Table 4:** Appraisal of ethical challenges in the decision-making process for VS patients

	**Median (1**^**st**^**, 3**^**rd **^**quartile) on NRS (0-10)**						
	All groups* (n = 332)	**Rank order**	Canadian group* (n = 200)	**Rank order**	German group* (n = 132)	**Rank order**	***p *** **value**
Determining patient’s wishes (n = 320)	7 (5,9)	1	7 (4.5,8)	2	8 (7,10)	1	p < 0.001
Accompanying family members in decisions (n = 322)	7 (5,8)	2	6 (3,8)	4	7 (6,9)	3	p < 0.001
Finding long-term care (n = 314)	7 (4,9)	3	8 (4,9)	1	6 (4,8)	6	p = 0.007
Making prognosis and predicting recovery (n = 323)	7 (4,9)	3	7 (3,8)	3	8 (7,10)	1	p < 0.001
Evaluating resource allocation (n = 318)	7 (3,9)	4	6 (3,8)	4	7 (4,9)	4	p = 0.010
Deciding for patient in absence of surrogate (n = 321)	7 (4,9)	4	5 (2,7)	6	8 (7,10)	1	p < 0.001
Assessing medical futility (n = 319)	6 (3,8)	5	4 (2,7)	7	7 (5,8)	5	p < 0.001
Making correct diagnosis (n = 325)	6 (2,8)	6	5 (2,8)	5	7 (4,9)	4	p < 0.001
Discontinuing LST (n = 323)	6 (2,8)	6	4 (2,7)	7	8 (5,10)	2	p < 0.001
Accompanying clients through staff rotations (n = 313)	5 (3,7)	7	5 (2,7)	6	6 (3.5,8)	7	p = 0.002
Applying a decision made by surrogate (n = 318)	5 (3,7)	7	4 (2,6)	8	7 (5,8)	5	p < 0.001
Reaching an agreement as a team (n = 320)	4 (2,6)	8	3 (2,5)	10	5 (3,7)	9	p < 0.001
Multidisciplinary discussions for decisions (n = 321)	3 (1,5)	9	2 (1,4)	9	5 (3,7)	8	p < 0.001

*Those who assigned the correct diagnosis to the patient in the case; Mann-Whitney-*U* test; average rank order according to Median, 1^st^ and 3^rd^ quartile on NRS (0-10).

## Discussion

This study aimed to assess similarities and differences in Canadian and German specialty physicians’ medical knowledge of the VS and attitudes toward ethical challenges in this disorder. This was the first such survey involving Canadian physicians. We found nearly identical rates of 80% for diagnostic accuracy in both samples and the subsamples of neurologists did not show greater accuracy rates than other physicians. Participants in both countries attributed a range of capabilities to patients in VS. The majority considered acceptable to limit life-sustaining treatment under certain circumstances. However, participants’ appraisals of ethical challenges differed between the countries.

Our findings indicate lower inaccuracy rates than the study by Schnakers and colleagues, who showed that 40% of the VS patients were misdiagnosed by doctors who had not used validated behavioral test instruments [6]. That the majority of physicians who provided a wrong diagnosis were certain of their answer in our study, raises the question of whether this group would perceive a need for further training in this area as previously recommended [20]. One of the most surprising findings of our study is the high proportion of participants in Canada and Germany who attributed capabilities to a patient in the VS akin to misattribution of capabilities observed in the public domain [21] (but contra Kickman and Wegner [22]). The key assumption underlying the traditional diagnosis of VS is the absence of awareness [23]. However, a majority of participants disagreed over whether the patient could perceive pain, and a majority of German participants and a large proportion of Canadian participants disagreed over whether the patient could feel touch. These results are not unprecedented [12,17,24,25], even if they sharply contrast to the medical understanding of the VS that is supported by fMRI research [26,27]. The differences between the two samples could be explained by the higher proportion of participants with religious or spiritual beliefs in the German sample given studies that have reported effects of religious beliefs in the care of patients with disorders of consciousness [12,13]. Another explanation might be that more German participants provide long-term care in the out-patient care setting and therefor might be able to observe more body expressions by patients in the VS in a rehabilitation process that could let them assume that such patients display capabilities inconsistent with common understandings of the diagnosis. The higher attribution of capabilities might lead to a higher reluctance to withdraw LST for patients in the VS.

Most Canadian participants identified long-term care placement as the most ethically challenging issue, perhaps because such facilities are rare in Canada. In a qualitative study, this issue and resource allocation were found to be important challenges in Canada [28]. Finding long-term-care placement and evaluating resource allocation were not perceived as challenging by German participants and a potential explanation lies in a system in which patients have greater access to long-term care facilities such as nursing homes, specialized centers for patients with disorders of consciousness (rehabilitation phase F, long term care to maintain function, delivered in specialized units), and nursing homes for patients who require artificial respiration [29,30]. These results are consistent with practice variations on different treatment measures reported previously [7,31-33]. They also suggest the potential impact of institutional medical practice, health care system, legal regulations and religion, as factors influencing participants’ attitudes toward treatment limitation and ethical challenges for patients in the VS as found, for example, in large-scale studies of LST decisions in Europe [34,35]. Although we can only speculate on the reasons for such variation (e.g., different culturally held moral traditions in either country) more Canadian participants favored always ceasing LST. One hypothesis, in line with recent research on the duality of moral theories [36], is that the attitudes of German participants, more reluctant to withdrawal of LST, may stem from a deontological philosophical tradition, where duties, rights and categorical principles of action have greater influence. The more rationalistic, utilitarian responses of Canadian participants may have led them to be more in favor of treatment limitation when the prognosis is unfavorable and chances for recovery are low. More specific data (e.g., on the preferred ethical theories of physicians in both countries) would be needed to test this hypothesis. A different explanation might link the history of serious ethical faults of the German medical profession under the Nazi regime [37] to the expression of prudental attitudes towards withdrawal of treatment for patients with severe disabilities. Acknowledging the existence of variation and seeking a clearer understanding of its causes are important steps to offer more coherent messages to family members and the public and to ensure a fair provision of treatment for patients with disorders of consciousness.

### Limitations

There was a small discrepancy in the case vignette (modification of one word, “consistently”, in the paper version of the Canadian questionnaire) for the sake of improved clarity. Our analysis of the Canadian data showed that it had no significant influence on the accuracy rate. Generalizability of the results of this long survey is limited by a focus on a single clinical condition and by a low response rate, especially in Canada, but we were able to gather representative samples of both German neurologists and Canadian physicians based on demographic variables. Individuals most likely excluded themselves from participation when they did not provide care for patients in the VS. The composition of the initial two samples differed in age, specialties represented, experience and physicians work setting, but the distributions of the experience with patients in the VS and gender were similar. These constitutive differences in the two cohorts could have influenced the described differences in the results. However, we compared subsamples of neurologists and found no differences in their accuracy rates. The original study design (e.g., recruitment strategy) was the same in both countries. Differences reported occurred because of adaptation to specific regulatory and institutional environments in the countries, such as policies of professional societies, availability of physicians’ addresses and willingness to respond.

## Conclusions

This survey of German and Canadian specialty physicians compared their understanding of and attitudes toward the VS. We found striking similarities in the participants’ medical knowledge with high diagnostic accuracy rates. However, we found important differences in the attribution of capabilities to the patients and attitudes toward limiting LST. Different hypotheses could explain this difference such as societal and medical practice contexts (e.g., distribution of resources for the long-term-care for these patients), religiosity, and underlying moral theories.

## Competing interests

The authors declare that they have no competing interests.

## Authors’ contributions

The final manuscript has been read and approved by all the authors and the requirements for authorship have been met by all authors, and each author believes that the manuscript represents honest work. All co-authors contributed to the original conception and design as well as the interpretation of the data. Acquisition of the data was handled by P, K, R, and J. K and R drafted the paper and all co-authors revised it critically for important intellectual content and gave final approval for submission to *BMC Neurology*.

## Pre-publication history

The pre-publication history for this paper can be accessed here:

http://www.biomedcentral.com/1471-2377/14/119/prepub

## Supplementary Material

Additional file 1: Table S1 Circumstances justifying the limitation of life-sustaining treatment. Click here for file
