# Supplementary material for: Physicians’ attitudes toward medical and ethical challenges for patients in the vegetative state: comparing Canadian and German perspectives in a vignette survey
Source: BMC Neurol. 2014 Jun 5;14:119. doi: 10.1186/1471-2377-14-119 (PMC4064260; doi:10.1186/1471-2377-14-119)
Supplement: Additional file 1: Table S1 — Circumstances justifying the limitation of life-sustaining treatment. [file 1471-2377-14-119-S1.docx]

**Additional file 1: Table S1: circumstances justifying the limitation of life-sustaining treatment.**

|  | **No (%)** | | |  | |
| --- | --- | --- | --- | --- | --- |
|  | All groups (n=332) | Canadian group (n=200) | German group (n=132) | | *p* value |
| **Patient’s will is opposed to LST (n=295)** | | | | | **p=0.076** |
| 5= extremely strong | 261 (89) | 163 (91) | 98 (85) | |  |
| 4 | 27 (9) | 14 (8) | 13 (11) | |  |
| 3 | 4 (1) | 1 (1) | 3 (3) | |  |
| 2 | 1 (0.3) | - | 1 (1) | |  |
| 1= extremely weak | 2 (1) | 1 (1) | 1 (1) | |  |
| **Patient suffers fatal disease (n=299)** | | | | | **p=0.671** |
| 5= extremely strong | 207 (69) | 127 (70) | 80 (68) | |  |
| 4 | 62 (21) | 39 (21) | 23 (20) | |  |
| 3 | 15 (5) | 9 (5) | 6 (5) | |  |
| 2 | 9 (3) | 4 (2) | 5 (4) | |  |
| 1= extremely weak | 6 (2) | 3 (2) | 3 (3) | |  |
| **Surrogate refuses consent to LST (n=298)** | | | | | **p<0.001** |
| 5= extremely strong | 165 (55) | 125 (69) | 40 (35) | |  |
| 4 | 72 (24) | 36 (20) | 36 (31) | |  |
| 3 | 42 (14) | 16 (9) | 26 (22) | |  |
| 2 | 15 (5) | 2 (1) | 13 (11) | |  |
| 1= extremely weak | 4 (1) | 3 (2) | 1 (1) | |  |
| **No chance: recovery of consciousness (n=292)** | | | | | **p<0.001** |
| 5= extremely strong | 173 (60) | 125 (70) | 48 (43) | |  |
| 4 | 66 (23) | 41 (23) | 25 (23) | |  |
| 3 | 24 (8) | 7 (4) | 17 (15) | |  |
| 2 | 23 (8) | 8 (4) | 15 (14) | |  |
| 1= extremely weak | 6 (2) | - | 6 (5) | |  |
| **No improvement > 1 year (n=287)** | | | | | **p<0.001** |
| 5= extremely strong | 134 (47) | 102 (57) | 32 (30) | |  |
| 4 | 69 (24) | 44 (24) | 25 (23) | |  |
| 3 | 49 (17) | 22 (12) | 27 (25) | |  |
| 2 | 21 (7) | 9 (5) | 12 (11) | |  |
| 1= extremely weak | 14 (5) | 3 (2) | 11 (10) | |  |
| **No chance: recovery of communication (n=290)** | | | | | **p=0.005** |
| 5= extremely strong | 78 (27) | 56 (31) | 22 (20) | |  |
|  | 88 (30) | 59 (32) | 29 (27) | |  |
| 3 | 69 (24) | 40 (22) | 29 (27) | |  |
| 2 | 39 (13) | 19 (10) | 20 (19) | |  |
| 1= extremely weak | 16 (6) | 8 (4) | 8 (7) | |  |
| **Patient obviously suffers intensely (n=286)** | | | | | **p<0.001** |
| 5= extremely strong | 109 (38) | 90 (51) | 19 (17) | |  |
| 4 | 75 (26) | 44 (25) | 31 (28) | |  |
| 3 | 62 (22) | 28 (16) | 34 (31) | |  |
| 2 | 21 (7) | 5 (3) | 16 (15) | |  |
| 1= extremely weak | 19 (7) | 10 (6) | 9 (8) | |  |
| **Patient is elderly (n=279)** | | | | | **p=0.002** |
| 5= extremely strong | 37 (13) | 30 (17) | 7 (7) | |  |
| 4 | 64 (23) | 47 (27) | 17 (17) | |  |
| 3 | 75 (27) | 44 (25) | 31 (30) | |  |
| 2 | 59 (21) | 30 (17) | 29 (28) | |  |
| 1= extremely weak | 44 (16) | 26 (15) | 18 (18) | |  |
| **No chance: recovery without disability (n=285)** | | | | | **p<0.001** |
| 5= extremely strong | 31 (11) | 25 (14) | 6 (6) | |  |
| 4 | 39 (14) | 30 (17) | 9 (9) | |  |
| 3 | 71 (25) | 56 (31) | 15 (15) | |  |
| 2 | 61 (21) | 33 (18) | 28 (27) | |  |
| 1= extremely weak | 83 (29) | 38 (21) | 45 (44) | |  |
| **Resources scarce and costs high (n=272)** | | | | | **p<0.001** |
| 5= extremely strong | 23 (9) | 21 (12) | 2 (2) | |  |
| 4 | 30 (11) | 25 (15) | 5 (5) | |  |
| 3 | 68 (25) | 51 (30) | 17 (17) | |  |
| 2 | 65 (24) | 35 (20) | 30 (30) | |  |
| 1= extremely weak | 86 (32) | 40 (23) | 46 (46) | |  |

Agreement to LST given certain circumstances; attitudes of participants who assigned the correct diagnosis to the vignette; Mann-Whitney U-Test
